# A computational approach to candidate gene prioritization for X-linked mental retardation using annotation-based binary filtering and motif-based linear discriminatory analysis

**DOI:** 10.1186/1745-6150-6-30

**Published:** 2011-06-13

**Authors:** Zané Lombard, Chungoo Park, Kateryna D Makova, Michèle Ramsay

**Affiliations:** 1 Division of Human Genetics, School of Pathology, Faculty of Health Sciences, National Health Laboratory Service & University of the Witwatersrand, Johannesburg, 2000, South Africa; 2 Center for Comparative Genomics and Bioinformatics, Department of Biology, The Pennsylvania State University, University Park, PA 16802, USA; 3 Department of Ecology and Evolutionary Biology, University of Michigan, 1075 Kraus Natural Science Bldg., 830 N. University, Ann Arbor, MI 48109-1048, USA

## Abstract

**Background:**

Several computational candidate gene selection and prioritization methods have recently been developed. These *in silico *selection and prioritization techniques are usually based on two central approaches - the examination of similarities to known disease genes and/or the evaluation of functional annotation of genes. Each of these approaches has its own caveats. Here we employ a previously described method of candidate gene prioritization based mainly on gene annotation, in accompaniment with a technique based on the evaluation of pertinent sequence motifs or signatures, in an attempt to refine the gene prioritization approach. We apply this approach to X-linked mental retardation (XLMR), a group of heterogeneous disorders for which some of the underlying genetics is known.

**Results:**

The gene annotation-based binary filtering method yielded a ranked list of putative XLMR candidate genes with good plausibility of being associated with the development of mental retardation. In parallel, a motif finding approach based on linear discriminatory analysis (LDA) was employed to identify short sequence patterns that may discriminate XLMR from non-XLMR genes. High rates (>80%) of correct classification was achieved, suggesting that the identification of these motifs effectively captures genomic signals associated with XLMR vs. non-XLMR genes. The computational tools developed for the motif-based LDA is integrated into the freely available genomic analysis portal Galaxy (http://main.g2.bx.psu.edu/). Nine genes (*APLN*, *ZC4H2*, *MAGED4*, *MAGED4B*, *RAP2C*, *FAM156A*, *FAM156B*, *TBL1X*, and *UXT*) were highlighted as highly-ranked XLMR methods.

**Conclusions:**

The combination of gene annotation information and sequence motif-orientated computational candidate gene prediction methods highlight an added benefit in generating a list of plausible candidate genes, as has been demonstrated for XLMR.

Reviewers: This article was reviewed by Dr Barbara Bardoni (nominated by Prof Juergen Brosius); Prof Neil Smalheiser and Dr Dustin Holloway (nominated by Prof Charles DeLisi).

## Background

The identification of genes and genetic variants that result in disease, or contribute to disease susceptibility, is a critical objective in medical research. Such findings have contributed to improvements in diagnosis, prognosis and therapy [1]. The typical approach taken for disease gene discovery for monogenic traits involves the identification of affected families, genotyping and linkage analysis. Subsequent fine mapping of the identified linked region is performed to focus the candidate region and reduce the number of putative candidate genes, and mutation detection is done to uncover the genetic cause of the disorder [2,3]. This approach has been successfully used to pinpoint the genetic contributors for over a thousand diseases, including Huntington disease [4], Duchenne muscular dystrophy [5] and cystic fibrosis [6]. With the successful identification of disease genes for many single-gene disorders, the focus has shifted to diseases with a complex, multifactorial etiology [7,8]. The candidate gene approach has often been used in the search for complex disease genes, but with the advent of massively parallel sequencing and genotyping, genome-wide approaches (such as genome-wide association studies [GWAS]) are starting to take precedence [9]. However, these approaches can result in large sets of potentially implicated genes, with the challenge then being to identify the actual genes involved with disease pathogenesis - a potentially laborious and costly exercise.

Recently, many computational candidate gene selection and prioritization methods have been developed, in part to provide new avenues to pinpoint disease genes or to prioritize genes from a large list of candidates [10-21]. Most often, one of two approaches is taken to identify and prioritize putative disease genes - either investigating similarities (such as sequence resemblance) to known disease genes or evaluating functional annotation of genes. The first is based on the premise that differences exist between disease genes, and other human genes, including differences in gene sequence and structure, which can then be utilized to single out candidate disease genes [13,14,22,23]. This information can also be used for other objectives such as the discovery of sequences important in X-chromosome inactivation, and the subsequent prediction of expression status of individual genes [24]. The second is an annotation-based approach that centers on the hypothesis that similar diseases may be influenced by genes with comparable features (such as function). This method relies heavily on the terms used to describe genes and their related products, and standardized ontologies utilized across databases (such as Gene Ontology (GO) and eVOC) are imperative to the efficiency of this approach.

In an attempt to increase the probability of correctly identifying disease related genes by producing a short list of highly probable candidate genes, a combined approach could reduce uncertainty. This could be done by combining multiple independent lines of evidence, each by itself lacking sufficient power. In this paper, a previously described method of candidate gene selection based primarily on gene annotation [25] is complemented by the evaluation of pertinent DNA sequence motifs or signatures to refine the gene selection approach [24]. The intercept between these approaches is evaluated, and the usefulness of combining the two is discussed. We have applied this approach to X-linked mental retardation (XLMR), a group of related but heterogeneous disorders for which some of the underlying genetics is known (reviewed in [26]).

## Results

### Annotation-based gene prioritization using a binary filtering method

**Figure 1:**
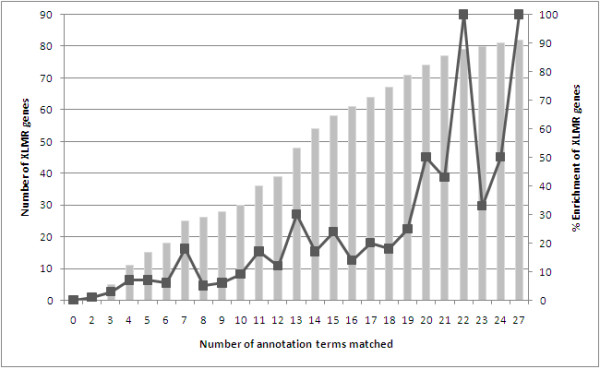
**Prioritization of genes on the X chromosome as XLMR candidates using a binary filtering process**. The effective cumulative coverage of XLMR genes from lowest to highest ranked categories is depicted (left Y-axis) as well as the percentage of genes that are XLMR-linked within each of the categories (right Y-axis).

The complete set of X chromosome genes (a total of 814 X-linked genes; Ensembl v49) was subjected to candidate gene selection for XLMR using a previously described method [25]. Gene annotation terms found to be relevant to XLMR were identified through literature and data-mining (a total of 40 terms; summarized in *Table *1). Each term was used as a selection criterion to populate a gene list (containing all genes within the Ensembl database annotated with that term). Candidate genes were then prioritized using a binary evaluation grid that assessed the term gene lists against the X-linked gene list, and genes were scored (see Methods for details) accordingly, and the X-linked gene list was subsequently prioritized for putative involvement in XLMR. X-linked genes with the most matches to the annotation-derived gene lists (27/40 being the most matches for any gene) were ranked as strong candidate XLMR genes. *Figure *1 shows the relative enrichment for known XLMR genes within the prioritized list, both cumulatively and as percentage enrichment. *Table S1 *(Additional File 1) shows the prioritization of X-linked genes as XLMR candidates using this approach, serving as a test for the sensitivity of this approach. The genes ranked highly by this method (i.e. more terms matched, therefore stronger support of being a likely candidate) have a higher enrichment for known XLMR genes than for non-XLMR genes. Conversely, the enrichment becomes moderated as one moves lower down the prioritized list.

**Table 1:** Annotation terms identified to be pertinent to XLMR using a literature- and data-mining approach

ANNOTATION TERM CATEGORIES^1^			
**Anatomical site**	**Biological Process**	**Phenotype**	**Animal model homology**

Developmental	Development	Seizures	*Phenotype*
Liver	Transcription	Epilepsy	Behaviour/Neurological
Central nervous system	Metabolism	Acidosis	Nervous system related
Respiratory	Phosphorylation	Microcephaly	Embryogenesis
Cerebellum	Brain development	Tremor	
Kidney			*Timing*
Hippocampus			Pre-Embryonic
Spinal cord			Embryonic
Cerebral cortex			Fetal
Testis			
Brain stem			*Anatomy*
Peripheral nerve			^2^TS8-9 Ectoderm
Cerebrum			TS10-13 Neural Ectoderm
Substantia nigra			TS14-26 CNS
Cardiovascular			
Adrenal gland			
Thyroid			
Ovary			
Amygdala			
Musculoskeletal			
Ganglion			
Hypothalamus			

^1^These terms were used to extract gene lists that were compared to all X chromosome genes in a binary filtering process. Annotation terms are divided into four categories based on their ontological classification.

^2^TS - Theiller stage: A term used to denote the stage of development of a mouse as described by Theiler in "The House Mouse: Atlas of Mouse Development" (Springer-Verlag, New York, 1989)

By considering the higher ranking genes on the prioritized list (signified by an arbitrary cut-off of 10/40 matches), it is observed that 66% (54/82) of known XLMR genes are present. This signifies a two-fold enrichment for XLMR genes (54/255; 0.212) in this group compared to all currently identified XLMR genes relative to X-linked genes (82/814; 0.101). Among the higher ranking genes, there were 201 genes that have not previously been associated with XLMR, which may be interesting candidates (*Table S2*, Additional File 1).

*MECP2*, a known XLMR causative gene (OMIM *300005), had the most annotation term matches (27/40) and matched at least one term in all four nomenclature categories used in prioritization (i.e., anatomical site terms, biological process, animal homology, and phenotype; see Methods for details). *MECP2 *is a widely expressed transcriptional repressor and has two conserved functional domains, the methyl-CpG binding domain and the transcription repression domain [27]. *MECP2 *displays extreme allelic heterogeneity, with more than 100 different mutations in the *MECP2 *gene being described in patients with Rett Syndrome [28,29]. Further to this, *MECP2 *mutations have also been shown to produce non-syndromic male fatal neonatal encephalopathy, progressive spasticity and non-syndromic Angelman and Prader-Willi-like phenotypes [29-32]. Rett syndrome is a prime example of the locus heterogeneity associated with XLMR as *MECP2 *mutations account for only approximately 70-80% of cases, whereas locus heterogeneity is hypothesized to explain the occurrence of the syndrome among *MECP2 *negative cases [29]. It is therefore likely that other genes that have been prioritized by the binary ranking method may be genetic candidates for Rett syndrome (among other forms of mental retardation).

The dystrophin gene (*DMD*) was ranked second highest (24/40 matches) and mutations in this gene have been associated with X-linked mental retardation in some, but not all cases. *DMD *is one of the largest known genes, measuring 2.4 Mb, and was identified as the gene responsible for Duchenne (DMD) and Becker (BMD) muscular dystrophies. Dystrophin mRNA is present in brain tissue and is therefore thought to potentially contribute to mental retardation, as observed in some DMD patients [33]. Dystrophin in brain is transcribed from a different promoter to that used in muscle. Chelly et al. (1990) [34] demonstrated that the brain-type promoter of the dystrophin gene is highly specific to neurons.

### Prioritization based on sequence motifs

Gene sets were selected to identify distinguishing sequence-based features in the immediately upstream regions of the transcriptions start sites (TSSs) between the sets. The X-linked genes were divided into two groups: genes demonstrated to be involved in XLMR (prior validated and published research) and genes that have not been identified as being involved in XLMR (called non-XLMR - this group would include both genes not involved in XLMR and genes that have not yet been discovered to cause XLMR). In this part of the study, 81 and 486 genes were labeled as XLMR and non-XLMR genes, respectively. The remaining X-linked genes were excluded from the analysis because of the lack of annotated putative transcription start sites. To consider the effects of the observed nonrandom distribution of XLMR genes along the X chromosome [35,36] and the possible influence of distinct origins and evolutionary pressures on XLMR genes [37], genes were labeled as belonging to the X-added region (XAR; representing evolutionary young strata) or the X-conserved region (XCR; representing ancestral mammalian X), determined by their genomic location [38,39] and were analyzed separately (*Table *2).

**Table 2:** Number of genes considered for sequence-based prioritization

	XAR	XCR	TOTAL
XLMR	25	56	**81**
Non-XLMR	110	376	**486**
**TOTAL**	**135**	**432**	

To detect sequence motifs (hereafter called "oligomers") that may discriminate XLMR from non-XLMR genes (separately for XAR and XCR genes), genomic sequences (hereafter called "subgenomes") were compiled to form four subgenomes. They included regions upstream from the transcription start sites of each gene (hereafter called "contigs") and four variable distances were considered for the contigs: 5 kilobases (kb), 10 kb, 50 kb, and 100 kb upstream from the TSS of each gene. If a contig overlapped with a neighboring contig and both shared the same profile (XLMR or non-XLMR), the two were merged into a single, larger contig. If a contig overlapped with a contig from the opposite profile (e.g., XLMR and non-XLMR), then both contigs were discarded. Finally, if a contig from one profile overlapped with any part of a gene that was classified in the opposite profile, the contig was discarded (*Table S3 *- Additional File 1). Because the 3' portions of genes regulate mRNA stability and translational efficiency [40,41] and play an important role in development and disease [42,43], these regions might contain cis-acting sequences regulating XLMR genes. To address this possibility, we performed the same analysis using subgenomes involving regions both upstream and downstream from the TSSs of genes. We found no distinct difference between the two approaches in terms of classification success rates (data not shown). For this reason, we use the subgenomes derived from the upstream region of the TSSs of genes in the subsequent analysis.

To identify the overrepresented oligomers in each subgenome, the following criteria were considered: first, each oligomer was required to occur at least ten times in the subgenome (because each subgenome usually had at least 10 genes; *Table S3*) and second, to be retained for a particular subgenome (e.g. XLMR; XAR), the oligomer was required to occur five times more frequently in that subgenome compared to the alternative (e.g. non-XLMR, XAR; because the median value for the gene ratio between XLMR and non-XLMR subgenomes is ~5; *Table S3*). For the analysis described below, only 12-mers within 10 kb, 50 kb, and 100 kb of each gene were used as they had the highest total number of overrepresented oligomers (*Table S4 *- Additional File 1); too few overrepresented oligomers were found in the 5 kb subgenomes, so this scale was omitted from the further analysis (see Methods for details). Permutation tests were performed to evaluate the significance of the overrepresentation of oligomers (*Table S5 *- Additional File 1). Subsequently, overlapping (among different subgenomes) oligomers were merged into longer ones resulting in 268 (two from XLMR and 266 from non-XLMR) and 584 (11 from XLMR and 573 from non-XLMR) overrepresented oligomers found for XAR and XCR, respectively. Remarkably, the majority of these oligomers (about 97%) overlapped with known classes of interspersed elements. There was no clear difference in sequence classes between XLMR and non-XLMR. For overrepresented oligomers not mapping to the interspersed elements, they did not match known transcription factor binding motifs from the JASPAR CORE database [44].

**Figure 2:**
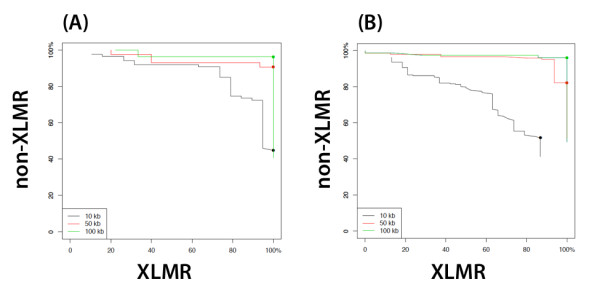
**LDA classification success rates for different values of the tuning parameter**. (A) All XAR genes were used for training and test sets. (B) All XCR genes were used for training and test sets. Leave-one-out cross-validation was utilized to calculate correct classification rates. Dots indicate optimal values of τ. More detailed information is given in *Table 3*.

Statistical analyses were performed to predict whether, using the set of overrepresented oligomers, genes could be classified as putative XLMR genes or non-XLMR genes. Linear discriminant analysis (LDA [45]) was used as a classifier to distinguish between XLMR and non-XLMR genes, considering XAR and XCR genes separately. Genes and counts of overrepresented oligomers upstream from TSSs of genes, identified above, were used as the units and features for classification. Utilizing two distinct sets of training data (either genes from the XAR or XCR), both LDA classifiers achieved classification accuracy of greater than 80% for both XLMR and non-XLMR classes except for the 10 kb range (*Table *3). The high rates of correct classification imply that classifiers based on counts of overrepresented oligomers effectively capture genomic signals found near XLMR vs. non-XLMR genes. In the case of the 100 kb range, correct classification rates for both classifiers were ≥ 96% for XLMR and non-XLMR classes (*Figure *2 and *Table *3). All genes tested in XAR with the 100 kb distance were perfectly classified (*Table S7 *- Additional File 1). For genes in XCR with the 100 kb distance, two genes (out of seven) in the XLMR class and three genes (out of 75) in non-XLMR class were incorrectly classified. Among these three incorrectly classified genes, one gene (*STARD8*) was consistently misclassified an XLMR gene at 10 kb and 50 kb distances as well, indicating that this gene may be a strong candidate XLMR gene. The other two genes that were misclassified with the 100 kb distance - *TRO *and *NUDT10 *- were not consistently misclassified across all three distances (Table S7), which lowers our confidence in them being true candidates. In addition to this there were a further seven non-XLMR genes that were incorrectly classified as XLMR-genes at both 10 kb and 50 kb distances (*ESX1, FGF16, ZC4H2, MAMLD1, ODZ1, PAGE4 *and *TMEM28)*.

**Table 3:** Number of genes for training and success rates of LDA

Set Analyzed	Parameter	10 kb (Genes)	50 kb (Genes)	100 kb (Genes)
Training and test set of genes in XAR	τ	0.96	0.43	0.4
	Success in XLMR	100% (19)	100% (15)	100% (9)
	Success in non-XLMR	45% (84)	91% (40)	96% (26)
Training and test set of genes in XCR	τ	0.87	0.75	0.62
	Success in XLMR	87% (38)	100% (16)	100% (7)
	Success in non-XLMR	52% (257)	82% (141)	96% (74)

τ is a tuning parameter, which was selected to maximize the sum of correct classification rates for XLMR and non-XLMR sets

### Combined analysis of annotation-based and sequence motif assessment in classifying X-linked genes as putative XLMR genes

To evaluate which genes are classified as high-probability XLMR genes by both of the assessment approaches, a step-wise approach was used. This was first done by analyzing the 255 top-ranked genes from the annotation-based approach (matching at least 10/40 criteria) and comparing them to the outcomes of the XLMR vs. non-XLMR discriminatory sequence-based classifiers. For this analysis, 154 genes (out of 255) were excluded from the dataset; either because of the lack of an annotated TSS (18 genes) or because of the short length of contigs (136 genes; namely, the distance from the TSS of the gene to its closest neighbor gene was less than 10 kb). The remaining 101 genes with at least 10 kb contigs were classified as XLMR [46] or non-XLMR genes and assessed using the sequence-based method to determine whether they had a XLMR or non-XLMR signature. In the case of genes whose contig length was greater than 50 kb (42 genes), 78.6% (33/42) had signatures as expected based on prior knowledge ("correctly classified" i.e. based on validated knowledge of involvement with XLMR vs. not previously associated with XLMR), and for contig lengths greater that 100 kb (22 genes), 86.4% (19/22) were correctly classified (*Table *4). Of particular interest would be the high-ranked candidates that had not previously been identified as being involved with XLMR, yet scored highly as probable XLMR genes and were therefore considered "incorrectly classified", i.e. they were included in the non-XLMR set, but may actually be XLMR genes. This group of genes would constitute good candidates for further research in the context of a mental retardation phenotype. Further analysis of genes with >50 kb contigs, using the sequence-based classifiers with the 50 kb and 100 kb distances, revealed that 14 genes were classified as putative XLMR genes (*Table *5). Five of them are known XLMR genes (*AP1S2*, *ARHGEF9*, *BCOR*, *HUWE1*, and *ZDHHC9*) and the remaining nine (*APLN*, *ZC4H2*, *MAGED4*, *MAGED4B*, *RAP2C*, *FAM156A*, *FAM156B*, *TBL1X*, and *UXT*) are strong candidates for XLMR and merit further analysis (*Table *6). Interestingly, only one of these nine genes (0.11) is located in the XAR, which is lower than the relative proportion of XAR genes analyzed (266/573; 0.46).

**Table 4:** Number of genes with ten or more matched categories using the annotation approach that were classified correctly by sequence-based LDA method

		Genes classified successfully		
		
Length of contigs	Number of genes tested	10 kb (Genes)	50 kb (Genes)	100 kb (Genes)
**> 10 kb**	101	54.5% (55)	52.5% (53)	52.5% (53)
**> 50 kb**	42	59.5% (25)	78.6% (33)	88.1% (37)
**> 100 kb**	22	59.1% (13)	81.8% (18)	86.4% (19)

**Table 5:** Classification of genes with > 50 kb contigs that were present in at least ten annotation categories

No of annotation terms matched (/40	HGNC symbol	XLMR gene^1^	50 kb^2^	100 kb^2^	Dist (kb)^3^	Strata
24	*DMD*	1	NX	NX	71.7	XAR
23	*GPM6B*	0	NX	NX	68	XAR
19	*AP1S2*	1	X	X	61.4	XAR
19	*FGF13*	0	NX	NX	157.8	XCR
19	*ZDHHC9*	1	X	NX	60.7	XCR
18	*TAF9B*	0	NX	NX	133	XCR
18	*FAM156A*	0	X	NX	90.8	XCR
18	*FAM156B*	0	X	NX	90.8	XCR
17	*ARHGEF9*	1	NX	X	259.5	XCR
17	*TMEM47*	0	NX	NX	285.5	XAR
16	*NAP1L2*	0	NX	NX	182.7	XCR
16	*NSBP1*	0	NX	NX	80.3	XCR
16	*PCSK1N*	0	NX	NX	56.7	XCR
16	*UXT*	0	X	NX	64.4	XCR
15	*BCOR*	1	X	X	253.8	XAR
15	*ENOX2*	0	NX	NX	155	XCR
15	*HUWE1*	1	X	NX	224.7	XCR
15	*LAS1L*	0	NX	NX	132.8	XCR
15	*THOC2*	0	NX	NX	124	XCR
14	*CXorf61*	0	NX	NX	323.4	XCR
14	*TBL1X*	0	X	NX	190.3	XAR
14	*TMLHE*	0	NX	NX	154.8	XCR
13	*C1GALT1C1*	0	NX	NX	102.9	XCR
13	*KAL1*	0	NX	NX	58.6	XAR
13	*PPP2R3B*	0	NX	NX	237.4	XAR
13	*RNF12*	0	NX	NX	65.4	XCR
13	*ZMAT1*	0	NX	NX	77	XCR
12	*GABRE*	0	NX	NX	103	XCR
12	*ZC4H2*	0	X	NX	431.8	XCR
12	*MAGED4*	0	NX	X	115.6	XCR
12	*MAGED4B*	0	NX	X	115.6	XCR
12	*MORC4*	0	NX	NX	64.2	XCR
12	*RAI2*	0	NX	NX	302.1	XAR
11	*APLN*	0	X	NX	84.5	XCR
11	*KLHL13*	0	NX	NX	83.1	XCR
11	*MUM1L1*	0	NX	NX	93.4	XCR
11	*RAP2C*	0	X	NX	160.1	XCR
11	*SLITRK4*	0	NX	NX	72.2	XCR
11	*SYTL4*	0	NX	NX	88.2	XCR
10	*CHRDL1*	0	NX	NX	300.5	XCR
10	*PHF16*	0	NX	NX	72.4	XAR
10	*STAG2*	0	NX	NX	60.9	XCR

^1^Indicates if gene is a known XLMR (= 1) or not (= 0)

^2^X indicates that a gene was classified as an XLMR gene and NX signifies classificiation as a non-XLMR gene.

^3^Distance from the TSS of gene to its closest neighbor gene

**Table 6:** Nine genes highlighted as XLMR candidates by both the annotation and sequence motif method

HGNC symbol	Description	Location	Function
*FAM156A*	Family with sequence similarity 156, member A	Xp11.23	Function Unknown
*FAM156B*	Family with sequence similarity 156, member B	Xp11.22	Function Unknown
*UXT*	Ubiquitously-expressed transcript	Xp11.23-p11.22	Plays a role in facilitating receptor-induced transcriptional activation [83]
*TBL1X*	Transducin (beta)-like 1X-linked	Xp22.3	Plays an essential role in transcription activation mediated by nuclear receptors [84]
*MAGED4*	Melanoma antigen family D, 4	Xp11	Mainly tumour cell proliferation [85]
*MAGED4B*	Melanoma antigen family D, 4B	Xp11	Mainly tumour cell proliferation [85]
*ZC4H2*	Zinc finger, C4H2 domain containing	Xq11.1	Hepatocellular carcinoma-associated antigen [38]
*APLN*	Apelin	Xq25	Neuropeptide involved in the regulation of body fluid homeostasis and cardiovascular functions [86]
*RAP2C*	Member of RAS oncogene family	Xq25	Involved in serum response element mediated gene transcription [64]

The second comparison was to examine all the non-XLMR genes that had a low predictive score on the sequence-based LDA within at least two categories (10 kb, 50 kb or 100 kb subgenomes) and to compare them to the scores based on the annotation-based binary filtering method. *STARD8 *was the only gene that was consistently misclassified as across all three distances. It did not receive a particularly high ranking using the annotation-based approach (6/40) and might have been overlooked as a putative XLMR gene by this method alone. The other two genes that were misclassified with the 100 kb distance - *TRO *and *NUDT10 *- were both highly ranked as candidates using the annotation method - receiving a score of 20/40 and 10/40 respectively. Of the other seven non-XLMR genes that were incorrectly classified at both 10 kb and 50 kb distances (*ESX1, FGF16, ZC4H2, MAMLD1, ODZ1, PAGE4 *and *TMEM28) *none received a high-rank score in the annotation method, and would have been missed if the candidates were evaluated by only this method.

## Discussion

Mental retardation is defined as a disability characterized by significant limitations both in intellectual functioning and adaptive behaviour [47]. The importance of genes on the X-chromosome in the cause of mental retardation has been recognized for decades, largely due to the fact that males outnumber females in nearly all surveys of mental retardation by approximately a third [48]. For the more than 200 forms of XLMR described, more than 82 causative genes have been catalogued [46] and ongoing research means that causative genes are continually identified [49-52].

Mammalian X and Y chromosomes diverged independently after evolving from a pair of autosomes [53-55]. The X chromosome is particularly gene-poor, has an overall low GC-content compared with the genome average [38] and is highly enriched for interspersed repeats. It can be divided into the following evolutionary domains: the X-added region (XAR) and the X-conserved region (XCR). In this study we use XLMR as an example to test a combined gene annotation and sequence-based assessment model for candidate disease gene prediction.

The annotation-based gene prioritization method using binary filtering identified 201 X-linked genes that were annotated in at least ten out of the 40 categories chosen as representing biological categories expected to have a link to XLMR, but which had not previously been associated with XLMR. The annotation categories included gene expression site, protein function, related phenotype and animal homology. Of the top ranked genes that had not previously been associated with a mental retardation phenotype, several are compelling candidates for XLMR based on their function.

The highest ranked novel candidate XLMR gene was *TIMP1 *(24/40 matches) that encodes a natural inhibitor of the matrix metalloproteinases (MMP), a group of peptidases involved in degradation of the extracellular matrix. *TIMP1 *was shown to reside within a genetic hotspot for neurodegenerative disorders on the X chromosome [56]. It has recently been shown that MMP9-mediated TIMP1-regulated extracellular proteolysis is a novel mechanism contributing to synaptic plasticity. It is thought that the MMP9/TIMP1 system could be involved in a broad range of physiological and pathological phenomena in various brain regions, including developmental reorganization of the cerebellum, hippocampus-dependent learning and long-term potentiation [57].

The major caveat of the annotation-based binary filtering method is that it relies on current gene annotation data in the public domain. These data are incomplete, but constantly growing, and therefore it is feasible that the method has a biased probability of prioritizing better annotated genes, regardless of whether these genes are relevant to the disease of interest.

In this study we used an additional evaluation method that depends on DNA sequence that is readily available in the public domain, and not on gene annotation, and would therefore be less biased. This method requires some information that would allow one to build training sets of genes to identify motifs that will discriminate between high probability candidate genes and weak candidates. We used a prioritization method based on sequence motifs to explore the feasibility of differentiating XLMR genes from non-XLMR genes. In order to construct the training data sets we needed to take into account the evolutionary origin of segments of the X chromosome, as explained above. It is known that the X chromosome can be divided into two evolutionary distinct domains, XAR and XCR. Therefore, for this investigation LDA classifiers were constructed for the XAR and XCR genes separately to consider the possible influence of distinct origins and evolutionary pressures on XLMR genes [37]. This showed high rates of correct classification for known XLMR genes and genes on the X chromosome not previously associated with XLMR. We tested whether a classifier trained on XAR genes could predict the XLMR status of XCR genes, and vice versa. A low success rate was achieved when each LDA classifier was used to predict the XLMR status in another test set (*Table S6 *and *Figure S1 *- Additional Files 1 and 2, respectively). It suggests that each set of overrepresented oligomers captures distinct genomic signals for XLMR genes in either the XAR or XCR, but not both.

Using the sequence-based approach, *STARD8 *(also known as *DLC3*), which is not a known XLMR gene, was identified as a strong candidate XLMR gene by the sequence-based classification approach. A partial deletion of *STARD8 *is associated with the craniofrontonasal syndrome especially in females [58]. *STARD8 *is also involved in the growth and metastasis of tumor cells [59] as are *DLC1 *and *DLC2*. Structurally, this protein is composed of three protein domains: a sterile α-motif, a RhoGAP (Rho GTPase-activating protein) domain, and a START domain (StAR, steroidogenic acute regulatory protein, related lipid transfer). King and colleagues (2002) [60] revealed that the StAR is expressed in glia and neurons of the mouse brain and has a role in the production of neurosteroids supporting *STARD8 *as a tentative biological role player in genetic disorders related to brain functioning.

One of the limitations of the sequence-based motif approach is the stipulation that a gene must have an annotated TSS and must have a region of at least 10 kb upstream of the TSS that does not overlap with another gene, in order to be assessed. This meant that out of the top 255 genes identified using the annotation-based binary filtering method, less than half (only 101) could be assessed.

Interestingly, *STARD8 *was not ranked within the top 255 genes using the annotation criteria as it only matched six of the 40 annotation criteria used for assessment. Of these criteria matches four were related to the anatomical site of expression (pinpointing the brain as a significant expression location of this gene) and the other two were related to phenotype similarity in the mouse. *STARD8 *is not a very extensively studied gene, which could explain its relatively low level of annotation and subsequent poor ranking obtained with the annotation-based evaluation

Despite these limitations, a set of nine genes not previously associated with XLMR has emerged as high likelihood candidates by both prioritization approaches. These genes represent the overlap of the highest ranking genes among those that could be assessed by both methods. A summary of these genes and their main functions is found in *Table *6. Evaluation of these genes' function and expression sites, among other features, reveal that these genes do have some putative link to mental retardation. First and most importantly, all of the genes are expressed in the central nervous system or brain in normal tissues and have all been linked to a neurological-related phenotype or function in the mouse model [61]. *APLN *has been shown to play a critical role in fluid homeostasis and pressure/volume homeostasis in the brain [62]. This particular function is of utmost importance to the developing brain and research has shown that *APLN *is involved in modifications of the microvasculature in the immature brain, affecting cerebral blood flow during a hypoxic insult [63]. *RAP2C *has a more general function as it forms part of the Ras family, which regulates a wide variety of cellular functions that include cell growth, differentiation, and apoptosis [64]. *TBLX1 *has previously been associated with lissencephaly, a rare brain formation disorder caused by defective neuronal migration resulting in a lack of brain fold and grooves development [65]. Similarly, *UXT *has been shown to interact with *RCAN1*, which codes for the Down syndrome candidate region 1 (DSCR1) protein, and it is thought to play a role in the mental disability features of this syndrome [66]. It is interesting that all nine of the prioritized genes also have a link to cancer. This could indicate an important role in expression regulation mechanisms such as epigenetic modifications, RNA interference and nonsense-mediated mRNA decay in XLMR development. These mechanisms are often disregulated in cancers, and show redundancy related to their role in human development and pathology [67-69].

One approach to evaluate the credibility of the genes prioritized by these two methods is to compare them to the set of XLMR genes recently identified through a large-scale resequencing project [51]. This study found six known XLMR genes (*AP1S2*, *CUL4B*, *BRWD3*, *UPF3B*, *ZDHHC9 *and *SLC9A6*) in which there were multiple truncating variants, as well as three (*SYP*, *ZNF711 *and *CASK*) that are new additions to the list of XLMR genes. Four of the known XLMR genes highlighted by the Tarpey et al. [51] study, with the exception of *BRWD3 *and *SLC9A6*, were also prioritized as top candidates by the annotation method, as were the three newly identified genes (*Table S2*). Due to exclusion criteria for the sequence-motif approach only one of the three newly identified XLMR genes was evaluated in that approach (*ZNF711*), and was not classified as a likely XLMR candidate.

A recent study [70] describes the prioritization of XLMR candidates based on spatially mapped gene expression, and focuses on the 718 genes on the X chromosome recently resequenced by Tarpey et al. [51]. There is significant overlap (*P *= 2.4 × 10^-6 ^by hypergeometric test: H(*k; N, m, n*), where *k *is intersection genes (33), *N *is the total number of genes tested (814), *m *is the number of genes targeted by [70] (56; from the best 10% of the prioritized lists), and *n *is the given XLMR genes (255)) between the prioritized genes from this paper and the ones prioritized by our annotation-based method (*Table S2*). It should be noted that this method also provides a novel list of candidates, highlighting the precision challenges of computational prediction methods, and underscores that the aim of these methods is to highlight promising candidates.

Although both methods described here have specific caveats, our approach does yield credible candidates for XLMR, and the merit of applying candidate gene prioritization methods with different areas of focus, in parallel, is demonstrated. Candidate gene prioritization for XLMR could be further improved by the addition of further lines of evidence, such as expression data, phylogenetic profiles or shared protein motifs.

## Conclusion

As information in public databases becomes more complete, gene annotation will improve, increasing the likelihood of detecting appropriate candidates and decreasing the bias due to uneven annotation of genes. When using a sequence motif-based approach examining regions upstream of the TSSs of genes, one is limited in compiling training sets, especially in regions that are gene rich, resulting in genome overlaps and leading to the exclusion of genes for analysis. More extensive sequenced-based analysis could investigate other gene regions, for example the 3'UTRs, for motifs that may be related to RNAi regulatory mechanisms [71]. While combining gene annotation information and sequence motif-orientated computational candidate gene prediction methods, it is possible to identify a set of plausible candidate genes for disease, as has been demonstrated for XLMR.

## Methods

### Annotation-based gene prioritization using binary filtering

A list of HGNC IDs for all genes on the X chromosome (n = 814) were obtained from Ensembl (v49) and were prioritized for XLMR candidature using a previously described computational method [25]. This is a gene prioritization method based on annotation and genes are evaluated to establish whether their known features indicate that they are likely to be involved in a particular disease. The main strength of this approach is the use of the eVOC ontology, which is a set of controlled vocabularies that merges gene expression data by linking genome sequence- and expression phenotype information. In particular, eVOC incorporates terms describing the sample source of cDNA, SAGE libraries and labeled target cDNAs for microarray experiments [72]. eVOC contains four orthogonal ontologies - anatomical system, cell type, pathology and developmental stage.

Annotation terms were divided into four categories based on their ontological classification (*Table *1) - anatomical site, function, phenotype and animal homology (no category takes precedence over another). Each term was then used as a selection criterion to populate a gene list (containing all genes within the Ensembl database annotated with that term). All X-linked genes were prioritized for XLMR using a binary evaluation grid. The most recent list of cloned XLMR genes (obtained from [46]) was compared to the ranked list to assess the ability of this approach to identify known XLMR genes. Gene annotation terms found to be pertinent to XLMR were identified through literature and data-mining as follows: PubMed abstracts related to XLMR were obtained and the literature mining tools Dragon Disease Explorer and Dragon TF Association Miner (DTFAM) [73] (Part of Dragon Explorer System (DES), a licensed tool of OrionCell http://www.orioncell.org) were used to extract eVOC and GO ontology terms, respectively from the abstracts. These terms were then used to populate the annotation term lists, as described above. The categories of genes that were selected from the mouse database are: genes associated with phenotypes relevant to XLMR; genes expressed at different prenatal developmental stages and genes expressed in the developing brain. To populate the genes list related to animal homology, human orthologues were obtained by mining the Jackson Laboratory Mouse Genome database [61].

The binary evaluation was performed as follows: A gene in the X-linked list was assigned a 1 when that gene was also present in an annotation term list. If the gene was absent from that list it was assigned a 0. For each of the X chromosome genes a final binary score was calculated, simply by summing all binary scores for each of the terms used. Then all genes were ranked based on this score, with those having higher scores being higher in the rank list. Genes in the list that matched most annotation terms (i.e. those genes that obtained the most 1-scores in the binary matrix) received the highest rank as XLMR candidates. Similarly, genes that matched very few or none of the terms have a lower rank and are considered to be weak candidates.

### Prioritization based on sequence motifs

To perform the analysis for sequence-based prioritization a total 87 non-redundant candidate XLMR genes were collected from four main sources: Euro-MRX consortium (http://www.euromrx.com/en/database.html; 18 genes), 83 genes from [74], 62 genes from [75] and 69 genes collected from additional experimental literature. Because it can be difficult to define the candidate non-XLMR genes, as many genes as possible that are likely involved in XLMR were considered. The majority of genes (80 out of 87) gathered here were already utilized in the annotation-based approach and seven candidate XLMR genes were additionally obtained. In this analysis, it was assumed that X-linked genes, excluding the 87 genes listed above, are non-XLMR genes, resulting in 647 X-linked genes being classified as non-XLMR genes. The reference sets of X-linked genes were obtained from the UCSC Refseq track (hg18) and the Ensembl HUGO track (release 48 of NCBI build 36).

The putative TSSs of genes were identified using the methods described in [76]. Briefly, using data from two sets of 5'-end-tag-capture technologies (i.e., CAGE [77] and PET [78]), TSS-tag clusters for genes were identified. To map the TSS-tag clusters to their corresponding genes, the following two criteria were considered. First, the strand of a TSS-tag cluster must be identical to the strand of a gene. Second, a TSS-tag cluster must be located in the 5' upstream region from the coding start site of a gene. If multiple TSS-tag clusters were identified for a single gene, then only the TSS-tag site supported by the highest number of tags was selected to be used as the representative TSS. If multiple TSS-tag clusters had the same highest tag score, the TSS cluster closest to the coding start site was chosen to serve as the representative TSS. To ensure the reliability of the TSS-tag data, TSS-tag clusters with a single tag were excluded. Using RefSeq [79], H-Invitational [80] and human ESTs [81] from the UCSC genome browser server (hg18), TSS-tag clusters were also discarded if the genomic coordinate of the 5' end of the putatively corresponding cDNAs or ESTs did not overlapped with the TSS-tag clusters. As a result, putative transcription start sites for 81 XLMR and 486 non-XLMR genes were obtained from experimental data.

The motif discovery method used in [24] was adopted to detect significantly overrepresented oligomers from each subgenome (i.e., the XLMR and non-XLMR subgenome), for the XAR and XCR genes separately. Briefly, first all possible oligomers found within each subgenome were enumerated and sequentially counted. Five specified sizes (8-, 12-, 16-, 20-, and 24-mers) of oligomers were considered. Counts of oligomers were combined with counts of their reverse complement sequences. Exact matches were required. To define an oligomer as an overrepresented oligomer, two criteria were used: the oligomer should (1) occur at least 10 times in the relevant subgenome and (2) should be enriched at least five-fold in the relevant subgenome as compared to the other subgenome (e.g. to be defined as an oligomer for XLMR using the 10 kb distance as an example, an oligomer must occur at least five times more often in the 10 kb region upstream of an XLMR gene vs. the frequency of the same oligomer in the 10 kb region upstream of a non-XLMR gene). Permutation tests were performed to evaluate whether the overrepresented oligomers identified were significantly overrepresented in one subgenome compared to the other. For this permutation test, the subgenomes (XLMR and non-XLMR subgenomes) were pooled together and then divided into nonoverlapping 2 kb fragments. Each 2 kb fragment was randomly assigned to either a pseudo-XLMR or a pseudo-non-XLMR subgenome until the two pseudo subgenomes were equal to the two actual subgenomes in size. Within each pseudo subgenome the oligomers that satisfied the above two criteria for overrepresented oligomers were identified. This process was repeated 1000 times, and those oligomers that were identified in fewer than 50 out of the 1000 pseudo subgenome trials (p < 0.05) were considered significantly overrepresented.

LDA analysis was performed as reported in [24] with the only difference being that the *P *value for the *p*-dimensional predictor vector was 268 for XAR and 584 for XCR classifiers (*Table *2). Leave-one-out cross-validation was utilized to calculate correct classification rates. The computational tools for Principal Component Analysis (PCA), LDA and ROC visualization were developed and integrated into a freely available genomic analysis portal, Galaxy (http://main.g2.bx.psu.edu/ under Statistics tools; [20]).

To evaluate the overrepresented oligomers for potential functional motifs, we compared them to known transcription factor binding sites. We aligned the oligomers against all binding sites in JASPAR CORE database (version 3.0) [44] using MATLIGN [82] with the Euclidean distance scoring function. RepeatMasker tables at the UCSC Genome Browser (hg18) were utilized to map the coordinates of transposable elements into the genomic region of X chromosome.

## Competing interests

The authors declare that they have no competing interests.

## Authors' contributions

MR proposed the initial idea, ZL did preliminary investigation, KDM stimulated the addition of the sequence motif-based analysis, and ZL and CP performed the bioinformatics analyses. MR, KDM, ZL and CP discussed and interpreted the results, ZL and CP wrote the first draft. All authors read and approved the final manuscript.

## Reviewers' comments

### Reviewer #1: Dr Barbara Bardoni (nominated by Prof Juergen Brosius)

Institute of Molecular and Cellular Pharmacology, University of Nice-Sophia Antipolis, Valbonne, France

This reviewer provided no comments for publication.

### Reviewer #2: Prof Neil Smalheiser

Department of Psychiatry, University of Illinois at Chicago, Chicago, IL, USA

This manuscript attempts to predict genes that can cause or contribute to X-linked mental retardation, by combining annotation-based and sequence-based methods. The underlying hypothesis is that genes which are "similar" to known causal genes will be likely to cause disease too. Although this is a very reasonable idea, the ms. is currently in very rough shape and the conclusions are misleading at best.

One problem is that the authors never address a fundamental question head-on: What is the expected/estimated total number of genes that they expect to exist that cause XLMR? What fraction is currently known, and what fraction of the remaining genes is similar to those already known? Maybe the vast majority of new genes to be discovered are NOT similar to those already known, so their method has quite limited power to discover. The gene numbers may be related to those given in Table S1, but they should be clearly discussed in the text at the outset.

Conversely, it is also possible (even likely) that ALL genes that are involved in synaptic function or signalling can cause XLMR when mutated or altered. If so, then again, their combined method has quite limited power and scope, and may show marginal improvement over simply making a list of all genes expressed at synapses.

There are also severe technical issues that should be resolved, if possible, which will be listed in order that they were encountered within the manuscript:

1. Their method excludes all noncoding RNAs, as well as elements (e.g., enhancers) whose effects are not local.

#### Authors' Response

Since the method is modular in its design, it could be adjusted to include any number of addition criteria. In this paper, we focus on the potential function of protein-coding genes, as a first-line of evaluation.

2. They acknowledge a "major caveat" in the annotation approach but this may actually be a fatal flaw. Namely, better studied genes are better annotated, and genes already known to be involved in disease are better studied. This probably creates a confound so that the authors cannot use the fact that known genes are highly ranked in their schemes as evidence that the method works well.

#### Authors' Response

This is correct; however, more genes are becoming well annotated. Although known disease genes are often well-studied, there are several other non-disease genes that are equally or even better studied. The premise here is that one expects genes involved in the same disease to share commonalities in their functional annotation, relative to other genes in the genome. Some types of annotation are less likely to be biased as they have been studies on a genome wide basis (i.e. gene expression site and animal homology). The method's modularity means that more evidence can be added to extend its accuracy and to strengthen the confidence in a finding (as is illustrated here by the addition of sequence motifs as an extra layer of evidence for candidacy).

3. On p. 8, they note that fully a third of known XLMR genes fail to be ranked highly by the annotation scheme, which indicates rather poor performance. An error analysis should be carried out to see why this is so, and whether the method can be improved [sequence based analysis does NOT improve performance since it was only applied to those genes that ranked highly by annotation alone].

#### Authors' Response

*The sequence-based approach was applied to the full gene set to produce a set of highly-ranked candidates. These are now listed in Table *2*and Table S7. This result was then compared to the highly-ranked list from the annotation approach (Table *4, 5, *and *6*). This approach has previously been shown to be appropriate for the investigation of complex disease aetiology due to selection of multiple most likely disease genes, and can be used to identify pathways and regulatory networks of aetiological significance.*

4. The genes not known experimentally to cause XLMR are treated like a negative set, but this is quite confusing, both technically and in the narrative of the paper. Really this is a mixed set (some truly negative, some truly positive but not yet discovered). It is very confusing to read that putative discoveries are those which are "incorrectly classified" in the non-XLMR set. How are those distinguished from errors in the method? What is the estimated relative numbers of true discoveries vs. errors in the "incorrectly classified" set?

#### Authors' Response

These sections have been clarified as follows:

"The X-linked genes were divided into two groups: genes demonstrated to be involved in XLMR (prior validated and published research) and genes that have not been identified as being involved in XLMR (called non-XLMR - this group would include both genes not involved in XLMR and genes that have not yet been discovered to cause XLMR)."

*And also "In the case of genes whose contig length was greater than 50 kb (42 genes), 78.6% (33/42) had signatures as expected based on prior knowledge ("correctly classified" i.e. based on validated knowledge of involvement with XLMR vs. not previously associated with XLMR), and for contig lengths greater that 100 kb (22 genes), 86.4% (19/22) were correctly classified (Table *4*). Of particular interest would be the high-ranked candidates that had not previously been identified as being involved with XLMR, yet scored highly as probable XLMR genes and were therefore considered "incorrectly classified", i.e. they were included in the non-XLMR set, but may actually be XLMR genes. This group of genes would constitute good candidates for further research in the context of a mental retardation phenotype."*

5. The sequence based approach is not very promising as used in this paper, both because it was only applied to a subset of genes [those that ranked highly by annotation] and because it could only be applied to a minority of THOSE genes. Even if the combined performance was perfect, it is not clear that this is a practical method that can be advocated for others to use and take seriously. As it is, the results do not clearly indicate how much better the combined method is, compared to each method alone, in the usual performance parameters (precision, recall, accuracy, etc.). The data may be there, but it was not obvious to me. I could not find a legend for Supplemental File 1.

#### Authors' Response

We did apply the sequence based approach for all 567 genes (81 and 486 for XLMR and non-XLMR genes, respectively; see p. 9-12). After we had done both analyses, we then scrutinized the 255 top-ranked genes from the annotation-based approach for their scoring in the motif-based method. We have now also added the inverse comparison.

We agree the reviewer that comparisons of the each method including combined one will be valuable, but this is not possible at present since we do not know which additional X-linked genes are currently unknown XLMR genes. However, our main purpose was to identify potential candidate XLMR genes using heterogeneous biological information.

The legend for Supplemental File 1 can be found at the end of this manuscript, under the heading of Description of additional data files.

6. In the list of references to prior work, Dmitar Hristovski and others who use implicit (not explicit) similarity should be added.

#### Authors' Response

These papers have been added.

### Reviewer #3: Dr Dustin Holloway (nominated by Prof Charles DeLisi)

Boston University Writing Program & Center for the History and Philosophy of Science, Boston University

#### 

##### General Remarks

It has been known for some time that the combination of orthogonal datatypes (e.g., motif data and functional annotation) produce results that are often better than individual methods at predicting the function of unannotated genes or suggesting their correlation to disease. However, the authors correctly point out that current GWAS studies are limited in pinpointing genes and mutations that are truly causative for disease. Here, the authors use a combination of data mining, sequence analysis, and pattern recognition algorithms to predict a set of genes that credibly relate to a specific disease, X-Linked Mental Retardation. Overall, their report is clear and very well written. The analysis performed, while not entirely novel was cleverly, methodically, and effectively applied to the specific problem of discovering candidate disease genes for a particular condition. I feel that their methods are valuable tools in the hunt for causative disease genes and should be applied to more disease conditions in the future. Though I would like to see some further issues discussed in greater detail (see below), and I would like to see a more complete analysis of discovered sequence motifs, I believe that their paper is of publishable quality and will be of significant value to the community.

##### Other Comments

• Results, paragraph 1: The authors use data mining to identify terms associated with XLMR and then use each of those terms to seed a gene list. The author's straightforward description doesn't place much emphasis on the fact that the ontology used to derive functional terms (e.g., eVOC) actually contains information from gene expression studies, sequence homology, etc. I believe that this information is important and increases the significance of their work. Some functional ontologies (e.g, ECC codes) operate on smaller scales and deal mainly with biochemical function. The author's chosen ontologies are broader and incorporate a variety of experimental data that are more relevant to the problem they are addressing.

#### Authors' Response

The following text was added, to emphasize the use of the eVOC ontologies in this approach (p23):

The main strength of this approach is the use of the eVOC ontology, which is a set of controlled vocabularies that merges gene expression data by linking genome sequence- and expression phenotype information. In particular, eVOC incorporates terms describing the sample source of cDNA, SAGE libraries and labeled target cDNAs for microarray experiments.

• Do they have a sense of how the varying types of data contribute to the analysis? That is, do sequence homology terms, gene expression terms, or GO terms tend to provide the most prediction power in their method? In future work, could the ontologies be improved? What scope do they have for adding additional datasets?

#### Authors' Response

The efficacy of varying types of data in prediction has not been tested on a test data set, but could be done in future, and the different prediction terms can then be weighted to include this knowledge. The scope of this paper was however not to test the proficiency of prediction of any one annotation type, but rather to assess whether increased efficiency in prediction is observed when adding more lines of evidence to this method.

• Methods used by the Authors rely to some extent on known functional annotations. In the annotation based analysis, known annotations are required to prioritize gene lists. In the motif analysis, known functions are required to identify positive and negative sets for motif discovery and LDA. However, there are presumably many additional candidate genes which are poorly annotated and don't contain useful transcriptional motifs and would thus be missed by these methods. The Authors have created a system which is simple enough and seems flexible enough to incorporate additional data. I would suggest a short discussion about how their method could be expanded to include new information and expand their ability to address genes of unknown function. For example, it would probably be simple to include phylogenetic profiles, or shared protein motifs (across species) to identify genes that may be related to disease. They touch on some ideas briefly, but this could be important future work for their method.

#### Authors' Response

As mentioned before, the method is modular in its design and can easily include any number of additions.

• In the section discussing "combined analysis" the authors' state "Interestingly only one of these nine genes is located in the XAR." What is the significance (or hypothesized significance) of this?

#### Authors' Response

The sentence was modified as follows: "Interestingly, only one of these nine genes (0.11) is located in the XAR, which is lower than the relative proportion of XAR genes analyzed (266/573; 0.46)."

• In the discussion, the authors discuss a reference (their reference 63) which uses spatially mapped gene expression to identify XLMR genes. The authors note that the gene set they prepared shows significant overlap with that of ref. 63. I suggest calculating the statistical significance of this overlap with a hypergeometric test. If significant, it would provide additional confidence that their method is enriching for interesting genes.

#### Authors' Response

We added the following to the manuscript:

*"There is significant overlap (P = 2.4e-6 by hypergeometric test: H(k; N, m, n), where k is intersection genes (33), N is the total number of genes tested (814), m is the number of genes targeted by *[70]*(56; from the best 10% of the prioritized lists), and n is the given XLMR genes (255)) between the prioritized genes from this paper and the ones prioritized by our annotation-based method (Table S2)."*

• For the motif analysis, the authors choose to examine 12 mers only. Why 12 mers specifically? What about known transcription factor motifs (e.g., Transfac motifs)?

#### Authors' Response

*We mentioned this in the manuscript (p. 11; "only 12-mers within 10 kb, 50 kb, and 100 kb of each gene were used as they had the highest total number of overrepresented oligomers (Table S4 - Additional File *1*)").*

To evaluate the overrepresented oligomers for potential functional motifs, we analysed them further and added the following to the manuscript:

"Remarkably, the majority of these oligomers (about 97%) overlapped with known classes of interspersed elements. There was no clear difference in sequence classes between XLMR and non-XLMR. For overrepresented oligomers not mapping to the interspersed elements, they did not match known transcription factor motifs from the JASPAR CORE database."

In addition: The method is included in the appropriate section.

• The authors identify several hundred over-represented oligomers in their datasets, but they don't comment on the sequences themselves. I think it is important to look at these sequences to decide how biologically relevant they might be. Are many of them similar, i.e., just variations of a few "core" motifs? Do they match with or correspond to any known mammalian transcription factor motifs? Do the motifs appear to be high in "information content," meaning that they appear to be biologically relevant rather than just repeat elements such as long stretches of GC or A sequence?

#### Authors' Response

These issues have been addressed above.

• While LDA is a perfectly valid method for classification, did the authors consider alternatives such as SVM or Random Forest? SVMs offer greater flexibility to choose non-linear classifiers and also have well established kernel methods to handle sequence motif data (e.g., the spectrum kernel: http://helix-web.stanford.edu/psb02/leslie.pdf ).

#### Authors' Response

While we agree that SVMs tend to offer greater flexibility than LDA (for example, any distributional assumptions of datasets (e.g., normal distribution) are not required, and continuous and qualitative variables in datasets are controlled well), however, if the main aim of the study is to extract many more variables (i.e., oligomers) including discriminative information (conversely, parsimonious and less variables for SVM) to classify the data as well as to achieve high classification accuracies, LDA fits well in this situation. Although we did not show them for our model in detail due to the small number of oligomers from XLMR, the use of the LDA has merit and would be worth performing as more XLMR genes become available.

## Supplementary Material

Additional File 1 **Supplementary tables supporting the analysis**. Click here for file

Additional File 2 **Figure S1 - LDA classification success rates for different values of the tuning parameter τ**. (A) Test set of XAR genes, with training performed on XCR genes. (B) Test set of XCR genes, with training performed on XAR genes. Leave-one-out cross-validation was utilized to calculate correct classification rates. Dots indicate optimal values of τ (see *Table S6 *- Additional file 1). Click here for file
