# Supplementary material for: A computational approach to candidate gene prioritization for X-linked mental retardation using annotation-based binary filtering and motif-based linear discriminatory analysis
Source: Biol Direct. 2011 Jun 13;6:30. doi: 10.1186/1745-6150-6-30 (PMC3142252; doi:10.1186/1745-6150-6-30)
Supplement: Additional File 1 — Supplementary tables supporting the analysis. [file 1745-6150-6-30-S1.PDF]

Table S1. Overview of the prioritization of genes on the X chromosome as XLMR candidates using a binary filtering process

No. of annotation terms matched (/40)	Total number of genes in category	Known XLMR genes in category	Non-XLMR genes in category	Category enrichment of XLMR genes (%)
27	1	1	0	100
24	2	1	1	50
23	3	1	2	33
22	2	2	0	100
21	7	3	4	43
20	6	3	3	50
19	16	4	12	25
18	17	3	14	18
17	15	3	12	20
16	21	3	18	14
15	17	4	13	24
14	35	6	29	17
13	30	9	21	30
12	25	3	22	12
11	35	6	29	17
10	23	2	21	9
9	36	2	34	6
8	22	1	21	5
7	40	7	33	18
6	49	3	46	6
5	55	4	51	7
4	81	6	75	7
3	93	3	90	3
2	182	2	180	1
0	1	0	1	0
**TOTAL**	**814**	**82**	**732**	

Genes were ranked as likely XLMR genes based on the number of annotation terms matched (more matches = more likely to be an XLMR gene).

Table S2. X-linked genes prioritized as putative XLMR genes using an annotation-based approach

# Binary matches	HGNC symbol	Known XLMR gene[1]	Ensembl ID	Description
27	*MECP2*	1	ENSG00000169057	Methyl cpg binding protein 2
24	*DMD*	1	ENSG00000198947	Dystrophin
24	*TIMP1*	0	ENSG00000102265	TIMP metallopeptidase inhibitor 1
23	*GPM6B*	0	ENSG00000046653	Glycoprotein M6B
23	*HDAC6*	0	ENSG00000094631	Histone deacetylase 6
23	*PLP1*	1	ENSG00000123560	Proteolipid protein 1
22	*ATRX*	1	ENSG00000085224	Alpha thalassemia/mental retardation syndrome X-linked
22	*SLC6A8*	1	ENSG00000130821	Solute carrier family 6
21	*ARAF*	0	ENSG00000078061	V-raf murine sarcoma 3611 viral oncogene homolog
21	*EIF2S3*	0	ENSG00000130741	Eukaryotic translation initiation factor 2, subunit 3 gamma, 52kda
21	*MAOA*	1	ENSG00000189221	Monoamine oxidase A
21	*PCTK1*	0	ENSG00000102225	Cyclin-dependent kinase 16
21	*PGRMC1*	0	ENSG00000101856	Progesterone receptor membrane component 1
21	*PQBP1*	1	ENSG00000102103	Polyglutamine binding protein 1
21	*TSPAN7*	1	ENSG00000156298	Tetraspanin 7
20	*CUL4B*	1	ENSG00000158290	Cullin 4B
20	*HPRT1*	1	ENSG00000165704	Hypoxanthine phosphoribosyltransferase 1
20	*MORF4L2*	0	ENSG00000123562	Mortality factor 4 like 2
20	*NGFRAP1*	0	ENSG00000166681	Nerve growth factor receptor
20	*PHF6*	1	ENSG00000156531	PHD finger protein 6
20	*TRO*	0	ENSG00000067445	Trophinin
19	*AP1S2*	1	ENSG00000182287	Adaptor-related protein complex 1, sigma 2 subunit
19	*BGN*	0	ENSG00000182492	Biglycan
19	*CXorf1*	0	ENSG00000221870	Chromosome X open reading frame 1
19	*FAM39DP*	0	ENSG00000185596	WAS protein family homolog 3 pseudogene
19	*FAM39E*	0	ENSG00000185596	WAS protein family homolog 1
19	*FGF13*	0	ENSG00000129682	Fibroblast growth factor 13
19	*FHL1*	0	ENSG00000022267	Four and a half LIM domains 1
19	*HTATSF1*	0	ENSG00000102241	HIV-1 Tat specific factor 1
19	*IDS*	1	ENSG00000010404	Iduronate 2-sulfatase
19	*LAMP2*	1	ENSG00000005893	Lysosomal-associated membrane protein 2
19	*MAGED1*	0	ENSG00000179222	Melanoma antigen family D, 1
19	*MAGED2*	0	ENSG00000102316	Melanoma antigen family D, 2
19	*NONO*	0	ENSG00000147140	Non-POU domain containing, octamer-binding
19	*PLS3*	0	ENSG00000102024	Plastin 3
19	*SAT1*	0	ENSG00000130066	Spermidine/spermine N1-acetyltransferase 1
19	*ZDHHC9*	1	ENSG00000188706	Zinc finger, DHHC-type containing 9

18	*AIFM1*	0	ENSG00000156709	Apoptosis-inducing factor, mitochondrion-associated, 1
18	*EFNB1*	0	ENSG00000090776	Ephrin-B1
18	*FLNA*	1	ENSG00000196924	Filamin A, alpha
18	*FUNDC2*	0	ENSG00000165775	FUN14 domain containing 2
18	*IDH3G*	0	ENSG00000067829	Isocitrate dehydrogenase 3
18	*JARID1C*	1	ENSG00000126012	Lysine
18	*MID1IP1*	0	ENSG00000165175	MID1 interacting protein 1
18	*MSL3L1*	0	ENSG00000005302	Male-specific lethal 3 homolog
18	*PDZD11*	0	ENSG00000120509	PDZ domain containing 11
18	*RBBP7*	0	ENSG00000102054	Retinoblastoma binding protein 7
18	*SFRS17A*	0	ENSG00000197976	Splicing factor, arginine/serine-rich 17A
18	*SYN1*	1	ENSG00000008056	Synapsin I
18	*TAF9B*	0	ENSG00000187325	TAF9B RNA polymerase II, TATA box binding protein
18	*TMEM29*	0	ENSG00000182646	Family with sequence similarity 156, member A
18	*TMEM29B*	0	ENSG00000179304	Family with sequence similarity 156, member B
18	*USP11*	0	ENSG00000102226	Ubiquitin specific peptidase 11
18	*WDR13*	0	ENSG00000101940	WD repeat domain 13
17	*ARHGEF9*	1	ENSG00000131089	Cdc42 guanine nucleotide exchange factor
17	*EBP*	0	ENSG00000147155	Emopamil binding protein
17	*L1CAM*	1	ENSG00000198910	L1 cell adhesion molecule
17	*NDUFB11*	0	ENSG00000147123	NADH dehydrogenase
17	*PJA1*	0	ENSG00000181191	Praja ring finger 1
17	*PSMD10*	0	ENSG00000101843	Proteasome
17	*RPL10*	1	ENSG00000147403	Small nucleolar RNA, H/ACA box 70
17	*STS*	0	ENSG00000101846	Steroid sulfatase
17	*TAZ*	0	ENSG00000102125	Tafazzin
17	*TFE3*	0	ENSG00000068323	Transcription factor binding to IGHM enhancer 3
17	*TMEM47*	0	ENSG00000147027	Transmembrane protein 47
17	*TSC22D3*	0	ENSG00000157514	TSC22 domain family, member 3
17	*UBE1*	0	ENSG00000130985	Ubiquitin-like modifier activating enzyme 1
17	*UBQLN2*	0	ENSG00000188021	Ubiquilin 2
17	*USP9X*	0	ENSG00000124486	Ubiquitin specific peptidase 9, X-linked
16	*AMOT*	0	ENSG00000126016	Angiomotin
16	*ARMCX2*	0	ENSG00000184867	Armadillo repeat containing, X-linked 2
16	*ATP11C*	0	ENSG00000101974	Atpase, class VI, type 11C
16	*CD99L2*	0	ENSG00000102181	CD99 molecule-like 2
16	*FAM127A*	0	ENSG00000134590	Family with sequence similarity 127, member A
16	*FAM3A*	0	ENSG00000071889	Family with sequence similarity 3, member A
16	*G6PD*	0	ENSG00000160211	Glucose-6-phosphate dehydrogenase
16	*MED14*	0	ENSG00000180182	Mediator complex subunit 14
16	*MSN*	0	ENSG00000147065	Moesin
16	*NAP1L2*	0	ENSG00000186462	Nucleosome assembly protein 1-like 2

16	*NRK*	0	ENSG00000123572	Nik related kinase
16	*NSBP1*	0	ENSG00000198157	High-mobility group nucleosome binding domain 5
16	*OCRL*	1	ENSG00000122126	Oculocerebrorenal syndrome of Lowe
16	*PCSK1N*	0	ENSG00000102109	Proprotein convertase subtilisin/kexin type 1 inhibitor
16	*SMC1A*	1	ENSG00000072501	Structural maintenance of chromosomes 1A
16	*SMS*	1	ENSG00000102172	Spermine synthase
16	*SSR4*	0	ENSG00000180879	Signal sequence receptor, delta
16	*UXT*	0	ENSG00000126756	Ubiquitously-expressed transcript
16	*VBP1*	0	ENSG00000155959	Von Hippel-Lindau binding protein 1
16	*ZBTB33*	0	ENSG00000177485	Zinc finger and BTB domain containing 33
16	*ZMYM3*	0	ENSG00000147130	Zinc finger, MYM-type 3
15	*ARHGEF6*	1	ENSG00000129675	Rac/Cdc42 guanine nucleotide exchange factor
15	*BCAP31*	0	ENSG00000185825	B-cell receptor-associated protein 31
15	*BCOR*	1	ENSG00000183337	BCL6 corepressor
15	*CASK*	0	ENSG00000147044	Calcium/calmodulin-dependent serine protein kinase
15	*ELF4*	0	ENSG00000102034	E74-like factor 4
15	*ENOX2*	0	ENSG00000165675	Ecto-NOX disulfide-thiol exchanger 2
15	*GPKOW*	0	ENSG00000068394	G patch domain and KOW motifs
15	*GPRASP1*	0	ENSG00000198932	G protein-coupled receptor associated sorting protein 1
15	*HUWE1*	1	ENSG00000086758	HECT, UBA and WWE domain containing 1
15	*LAS1L*	0	ENSG00000001497	LAS1-like
15	*MAGEH1*	0	ENSG00000187601	Melanoma antigen family H, 1
15	*PHF8*	1	ENSG00000172943	PHD finger protein 8
15	*PIN4*	0	ENSG00000102309	Protein
15	*RBM10*	0	ENSG00000182872	RNA binding motif protein 10
15	*RBM3*	0	ENSG00000102317	RNA binding motif
15	*SYBL1*	0	ENSG00000124333	Vesicle-associated membrane protein 7
15	*THOC2*	0	ENSG00000125676	THO complex 2
14	*ACSL4*	1	ENSG00000068366	Acyl-coa synthetase long-chain family member 4
14	*ARMCX3*	0	ENSG00000102401	Armadillo repeat containing, X-linked 3
14	*ARMCX6*	0	ENSG00000198960	Armadillo repeat containing, X-linked 6
14	*CAPN6*	0	ENSG00000077274	Calpain 6
14	*CITED1*	0	ENSG00000125931	Cbp/p300-interacting transactivator, with Glu/Asp-rich carboxy-terminal domain, 1
14	*CXorf61*	0	ENSG00000204019	Chromosome X open reading frame 61
14	*DCX*	1	ENSG00000077279	Doublecortin
14	*F8*	0	ENSG00000185010	Coagulation factor VIII, procoagulant component
14	*GJB1*	0	ENSG00000169562	Gap junction protein, beta 1, 32kda
14	*GNL3L*	0	ENSG00000130119	Guanine nucleotide binding protein-like 3
14	*GRIA3*	1	ENSG00000125675	Glutamate receptor, ionotrophic, AMPA 3

14	*GRIPAP1*	0	ENSG00000068400	GRIP1 associated protein 1
14	*GSPT2*	0	ENSG00000189369	G1 to S phase transition 2
14	*HCCS*	1	ENSG00000004961	Holocytochrome c synthase
14	*HCFC1*	0	ENSG00000172534	Host cell factor C1
14	*IGBP1*	0	ENSG00000089289	Immunoglobulin
14	*IL2RG*	0	ENSG00000147168	Interleukin 2 receptor, gamma
14	*MAP7D3*	0	ENSG00000129680	MAP7 domain containing 3
14	*MID1*	1	ENSG00000101871	Midline 1
14	*PCYT1B*	0	ENSG00000102230	Phosphate cytidylyltransferase 1, choline, beta
14	*PRKX*	0	ENSG00000183943	Protein kinase, X-linked
14	*RNF128*	0	ENSG00000133135	Ring finger protein 128
14	*RPL39*	0	ENSG00000198918	Ribosomal protein L39
14	*SCML1*	0	ENSG00000047634	Sex comb on midleg-like 1
14	*SH3BGRL*	0	ENSG00000131171	SH3 domain binding glutamic acid-rich protein like
14	*SLC25A14*	0	ENSG00000102078	Solute carrier family 25
14	*SLC35A2*	0	ENSG00000102100	Solute carrier family 35
14	*TAF1*	0	ENSG00000147133	TAF1 RNA polymerase II, TATA box binding protein
14	*TBL1X*	0	ENSG00000101849	Transducin
14	*TMEM32*	0	ENSG00000169446	Membrane magnesium transporter 1
14	*TMLHE*	0	ENSG00000185973	Trimethyllysine hydroxylase, epsilon
14	*UPF3B*	1	ENSG00000125351	UPF3 regulator of nonsense transcripts homolog B
14	*UTX*	0	ENSG00000147050	Lysine (K)-specific demethylase 6A
14	*ZNF185*	0	ENSG00000147394	Zinc finger protein 185
14	*ZRSR2*	0	ENSG00000169249	Zinc finger
13	*C1GALT1C1*	0	ENSG00000171155	C1GALT1-specific chaperone 1
13	*DNASE1L1*	0	ENSG00000013563	Deoxyribonuclease I-like 1
13	*ELK1*	0	ENSG00000126767	ELK1, member of ETS oncogene family
13	*FMR1*	1	ENSG00000102081	Fragile X mental retardation 1
13	*FTSJ1*	1	ENSG00000068438	Ftsj homolog 1
13	*GLA*	0	ENSG00000102393	Galactosidase, alpha
13	*HSD17B10*	1	ENSG00000072506	Hydroxysteroid
13	*IL3RA*	0	ENSG00000185291	Interleukin 3 receptor, alpha
13	*KAL1*	0	ENSG00000011201	Kallmann syndrome 1 sequence
13	*MED12*	1	ENSG00000184634	Mediator complex subunit 12
13	*MTM1*	1	ENSG00000171100	Myotubularin 1
13	*MXRA5*	0	ENSG00000101825	Matrix-remodelling associated 5
13	*NLGN3*	1	ENSG00000196338	Neuroligin 3
13	*NXT2*	0	ENSG00000101888	Nuclear transport factor 2-like export factor 2
13	*PDHA1*	1	ENSG00000131828	Pyruvate dehydrogenase
13	*PORCN*	1	ENSG00000102312	Porcupine homolog
13	*PPP2R3B*	0	ENSG00000167393	Protein phosphatase 2
13	*RNF12*	0	ENSG00000131263	Ring finger protein, LIM domain interacting
13	*RPS26L1*	0	ENSG00000196933	Ribosomal protein S26 pseudogene 11
13	*SEDLP*	0	ENSG00000196459	Trafficking protein particle complex 2 pseudogene 1

13	*SLC16A2*	1	ENSG00000147100	Solute carrier family 16, member 2
13	*SUV39H1*	0	ENSG00000101945	Suppressor of variegation 3-9 homolog 1
13	*TCEAL1*	0	ENSG00000172465	Transcription elongation factor A
13	*TCEAL8*	0	ENSG00000180964	Transcription elongation factor A
13	*TRAPPC2*	0	ENSG00000196459	Trafficking protein particle complex 2 pseudogene 1
13	*TREX2*	0	ENSG00000183479	Three prime repair exonuclease 2
13	*ZFX*	0	ENSG00000005889	Zinc finger protein, X-linked
13	*ZIC3*	0	ENSG00000156925	Zic family member 3
13	*ZMAT1*	0	ENSG00000166432	Zinc finger, matrin type 1
13	*ZNF449*	0	ENSG00000173275	Zinc finger protein 449
12	*ABCB7*	0	ENSG00000131269	ATP-binding cassette, sub-family B
12	*ALAS2*	0	ENSG00000158578	Aminolevulinate, delta-, synthase 2
12	*ARX*	1	ENSG00000004848	Aristaless related homeobox
12	*BCORL1*	0	ENSG00000085185	BCL6 corepressor-like 1
12	*CHST7*	0	ENSG00000147119	Carbohydrate
12	*DACH2*	0	ENSG00000126733	Dachshund homolog 2
12	*EGFL6*	0	ENSG00000198759	EGF-like-domain, multiple 6
12	*FAM58A*	0	ENSG00000147382	Family with sequence similarity 58, member A
12	*GABRE*	0	ENSG00000102287	Gamma-aminobutyric acid
12	*GLUD2*	0	ENSG00000182890	Glutamate dehydrogenase 2
12	*IKBKG*	1	ENSG00000073009	Inhibitor of kappa light polypeptide gene enhancer in B-cells, kinase gamma
12	*KIAA1166*	0	ENSG00000126970	Zinc finger, C4H2 domain containing
12	*MAGED4*	0	ENSG00000154545	Small nucleolar RNA, H/ACA box 11E
12	*MAGED4B*	0	ENSG00000187243	Small nucleolar RNA, H/ACA box 11E
12	*MAP3K15*	0	ENSG00000180815	Mitogen-activated protein kinase kinase kinase 15
12	*MORC4*	0	ENSG00000133131	MORC family CW-type zinc finger 4
12	*MTCP1*	0	ENSG00000214827	Mature T-cell proliferation 1
12	*PIGA*	0	ENSG00000165195	Phosphatidylinositol glycan anchor biosynthesis, class A
12	*RAI2*	0	ENSG00000131831	Retinoic acid induced 2
12	*RBMX2*	0	ENSG00000134597	RNA binding motif protein, X-linked 2
12	*RGN*	0	ENSG00000130988	Regucalcin
12	*SCML2*	0	ENSG00000102098	Sex comb on midleg-like 2
12	*SPIN2B*	0	ENSG00000186787	Spindlin family, member 2B
12	*TCEAL3*	0	ENSG00000196507	Transcription elongation factor A
12	*UBE2A*	1	ENSG00000077721	Ubiquitin-conjugating enzyme E2A
11	*ABCD1*	1	ENSG00000101986	ATP-binding cassette, sub-family D
11	*AFF2*	1	ENSG00000155966	AF4/FMR2 family, member 2
11	*APLN*	0	ENSG00000171388	Apelin
11	*ATG4A*	0	ENSG00000101844	ATG4 autophagy related 4 homolog A
11	*BMX*	0	ENSG00000102010	BMX non-receptor tyrosine kinase
11	*CTPS2*	0	ENSG00000047230	CTP synthase II
11	*CXorf41*	0	ENSG00000080572	Chromosome X open reading frame 41
11	*DLG3*	1	ENSG00000082458	Discs, large homolog 3
11	*DOCK11*	0	ENSG00000147251	Dedicator of cytokinesis 11
11	*DUSP9*	0	ENSG00000130829	Dual specificity phosphatase 9

11	*ERCC6L*	0	ENSG00000186871	Excision repair cross-complementing rodent repair deficiency, complementation group 6-like
11	*FAM50A*	0	ENSG00000071859	Family with sequence similarity 50, member A
11	*FIGF*	0	ENSG00000165197	C-fos induced growth factor
11	*FOXO4*	0	ENSG00000184481	Forkhead box O4
11	*GYG2*	0	ENSG00000056998	Glycogenin 2
11	*HEPH*	0	ENSG00000089472	Hephaestin
11	*IGSF1*	0	ENSG00000147255	Immunoglobulin superfamily, member 1
11	*KLHL13*	0	ENSG00000003096	Kelch-like 13
11	*KLHL15*	0	ENSG00000174010	Kelch-like 15
11	*MUM1L1*	0	ENSG00000157502	Melanoma associated antigen
11	*NLGN4X*	1	ENSG00000146938	Neuroligin 4, X-linked
11	*OFD1*	1	ENSG00000046651	Oral-facial-digital syndrome 1
11	*OTUD5*	0	ENSG00000068308	OTU domain containing 5
11	*PGAM4*	0	ENSG00000226784	Phosphoglycerate mutase family member 4
11	*PHKA1*	0	ENSG00000067177	Phosphorylase kinase, alpha 1
11	*PNCK*	0	ENSG00000130822	Pregnancy up-regulated non-ubiquitously expressed cam kinase
11	*RAB9A*	0	ENSG00000123595	RAB9A, member RAS oncogene family
11	*RAB9B*	0	ENSG00000123570	RAB9B, member RAS oncogene family
11	*RAP2C*	0	ENSG00000123728	RAP2C, member of RAS oncogene family
11	*SLITRK4*	0	ENSG00000179542	SLIT and NTRK-like family, member 4
11	*SYP*	0	ENSG00000102003	Synaptophysin
11	*SYTL4*	0	ENSG00000102362	Synaptotagmin-like 4
11	*TCEAL2*	0	ENSG00000184905	Transcription elongation factor A
11	*WDR45*	0	ENSG00000196998	WD repeat domain 45
11	*ZNF41*	1	ENSG00000147124	Zinc finger protein 41
10	*ALG13*	0	ENSG00000101901	Asparagine-linked glycosylation 13 homolog
10	*ATP2B3*	0	ENSG00000067842	Atpase, Ca++ transporting, plasma membrane 3
10	*CHRDL1*	0	ENSG00000101938	Chordin-like 1
10	*COL4A5*	0	ENSG00000188153	Collagen, type IV, alpha 5
10	*CXorf48*	0	ENSG00000169551	Chromosome X open reading frame 48
10	*DDX26B*	0	ENSG00000165359	Dead/h
10	*IL13RA2*	0	ENSG00000123496	Interleukin 13 receptor, alpha 2
10	*NUDT10*	0	ENSG00000122824	Nudix
10	*NXF2*	0	ENSG00000185554	Nuclear RNA export factor 2
10	*OPHN1*	1	ENSG00000079482	Oligophrenin 1
10	*PAK3*	1	ENSG00000077264	P21 protein
10	*PHF16*	0	ENSG00000102221	PHD finger protein 16
10	*PPP1R3F*	0	ENSG00000049769	Protein phosphatase 1, regulatory
10	*RRAGB*	0	ENSG00000083750	Ras-related GTP binding B
10	*SATL1*	0	ENSG00000184788	Spermidine/spermine N1-acetyl transferase-like 1
10	*SLC10A3*	0	ENSG00000126903	Solute carrier family 10
10	*SLITRK2*	0	ENSG00000185985	SLIT and NTRK-like family, member 2
10	*STAG2*	0	ENSG00000101972	Stromal antigen 2

10	*TMEM164*	0	ENSG00000157600	Transmembrane protein 164
10	*TSPYL2*	0	ENSG00000184205	TSPY-like 2
10	*WDR44*	0	ENSG00000131725	WD repeat domain 44
10	*ZCCHC12*	0	ENSG00000174460	Zinc finger, CCHC domain containing 12
10	*ZNF711*	0	ENSG00000147180	Zinc finger protein 711

[1]indicates if gene is a known XLMR (=1) or not (=0) (Chiurazzi et al., 2008)

Table S3. Number of genes in each subgenome

	X-added region				X-conserved region			
	XLMR		Non-XLMR		XLMR		Non-XLMR	
Contig size	# Genes	Length[1]	# Genes	Length[1]	# Genes	Length[1]	# Genes	Length[1]
5 kb	21	0.1	96	0.5	45	0.2	291	1.5
10 kb	19	0.2	84	0.9	38	0.4	257	2.7
50 kb	15	0.8	40	2.2	16	0.8	141	7.3
100 kb	9	0.9	26	2.7	7	0.7	74	7.7

[1]The length of each subgenome is given in megabases (Mb).

Table S4. Number of overrepresented oligomers in each subgenome

	X-added Region								X-conserved Region							
	XLMR				Non-XLMR				XLMR				Non-XLMR			
	Contig (kb)				Contig (kb)				Contig (kb)				Contig (kb)			
**Oligomers**	**5**	**10**	**50**	**100**	**5**	**10**	**50**	**100**	**5**	**10**	**50**	**100**	**5**	**10**	**50**	**100**
8-mers	9	4	6	22	757	411	160	136	0	2	2	1	753	819	889	1518
12-mers	3	6	72	68	167	314	686	860	3	29	25	9	700	3112	12660	20522
16-mers	0	0	69	71	157	247	309	536	1	8	8	14	317	2255	3710	6171
20-mers	0	0	56	56	89	199	188	357	0	0	2	10	199	1987	2804	4228
24-mers	0	0	37	37	64	124	120	230	0	0	1	4	145	1805	2044	3206

Table S5. Number of significantly overrepresented 12-mers with $P < 0.05$

	X-added region		X-conserved region	
	**XLMR**	**Non-XLMR**	**XLMR**	**Non-XLMR**
10 kb	3	44	6	625
50 kb	2	259	1	288
100 kb	2	259	9	622

Table S6. Number of genes for test sets and success rates of LDA

Set Analyzed	Parameter	10 kb (Genes)	50 kb (Genes)	100 kb (Genes)
Test set of genes in XCR	τ	0.95	0.93	0.8
using training set derived	Success in XLMR	74% (38)	63% (16)	43% (7)
from XAR genes	Success in non-XLMR	32% (257)	30% (141)	39% (74)
Test set of genes in XAR	τ	0.98	0.96	0.94
using training set derived	Success in XLMR	84% (19)	73% (15)	78% (9)
from XCR genes	Success in non-XLMR	32% (84)	37% (40)	48% (26)

τ is a tuning parameter, which was selected to maximize the sum of correct classification rates for XLMR and non-XLMR sets.

Table S7. Correctly and incorrectly classified genes using the identified oligomer signature

A **X-added Region**

Genes	Classes	10 kb	50 kb	100 kb
BCOR	XLMR	O	O	O
DMD	XLMR	O	O	O
HCCS	XLMR	O	O	O
IL1RAPL1	XLMR	O	O	O
MAOA	XLMR	O	O	O
PDHA1	XLMR	O	O	O
RPS6KA3	XLMR	O	O	O
TSPAN7	XLMR	O	O	O
VCX3A	XLMR	O	O	O
CD99	Non-XLMR	O	O	O
CNKSR2	Non-XLMR	X	O	O
DDX53	Non-XLMR	X	O	O
EGFL6	Non-XLMR	X	O	O
FAM47B	Non-XLMR	O	O	O
FAM9C	Non-XLMR	X	O	O
FUNDC1	Non-XLMR	O	O	O
GEMIN8	Non-XLMR	X	O	O
GLRA2	Non-XLMR	X	O	O
GPR64	Non-XLMR	O	O	O
GRPR	Non-XLMR	X	O	O
MAGEB10	Non-XLMR	O	O	O
MAGEB18	Non-XLMR	X	O	O
MAGEB2	Non-XLMR	X	O	O
MAP3K7IP3	Non-XLMR	X	O	O
MID1IP1	Non-XLMR	X	O	O
PDK3	Non-XLMR	O	O	O
PPP2R3B	Non-XLMR	O	X	O
PRDX4	Non-XLMR	X	O	O
PTCHD1	Non-XLMR	X	O	O
RAI2	Non-XLMR	X	O	O
TMEM47	Non-XLMR	X	O	O
USP9X	Non-XLMR	O	O	O
VCX	Non-XLMR	X	O	O
VCX2	Non-XLMR	X	O	O
VCX3B	Non-XLMR	X	O	O

B **X-Conserved Region**

Genes	Classes	10 kb	50 kb	100 kb
AGTR2	XLMR	O	O	O
ARHGEF9	XLMR	O	O	X
KIAA2022	XLMR	O	O	O
KLF8	XLMR	O	O	O
PAK3	XLMR	O	O	O
SOX3	XLMR	O	O	X
ZDHHC15	XLMR	O	O	O
ACTRT1	Non-XLMR	O	X	O
APOOL	Non-XLMR	O	O	O
AR	Non-XLMR	X	O	O
C1GALT1C1	Non-XLMR	O	O	O
CDR1	Non-XLMR	O	O	O
CHIC1	Non-XLMR	X	O	O
CHM	Non-XLMR	X	O	O
CHRDL1	Non-XLMR	X	O	O
CPXCR1	Non-XLMR	O	O	O
CXorf26	Non-XLMR	O	O	O
CXorf40B	Non-XLMR	X	O	O
CXorf57	Non-XLMR	O	O	O
CXorf61	Non-XLMR	O	O	O
CYSLTR1	Non-XLMR	O	O	O
DACH2	Non-XLMR	X	O	O
DIAPH2	Non-XLMR	X	O	O
ENOX2	Non-XLMR	X	O	O
ESX1	Non-XLMR	X	X	O
FGF13	Non-XLMR	X	O	O
FGF16	Non-XLMR	X	X	O
FXYD8	Non-XLMR	X	O	O
GABRE	Non-XLMR	O	O	O
GPC4	Non-XLMR	X	O	O
GSPT2	Non-XLMR	O	X	O
HDX	Non-XLMR	X	O	O
HEPH	Non-XLMR	O	O	O
IL1RAPL2	Non-XLMR	X	O	O
ITM2A	Non-XLMR	O	O	O
ZC4H2	Non-XLMR	X	X	O
KLHL4	Non-XLMR	O	O	O
LAS1L	Non-XLMR	X	O	O
LOC203547	Non-XLMR	O	O	O
LONRF3	Non-XLMR	X	O	O
MAGEA4	Non-XLMR	O	X	O
MAGEE2	Non-XLMR	O	O	O
MAGEH1	Non-XLMR	O	O	O
MAMLD1	Non-XLMR	X	X	O
MSN	Non-XLMR	X	O	O
NAP1L2	Non-XLMR	O	O	O
NKAP	Non-XLMR	O	O	O

NUDT10	Non-XLMR	X	O	X
ODZ1	Non-XLMR	X	X	O
PABPC5	Non-XLMR	O	O	O
PAGE1	Non-XLMR	X	O	O
PAGE4	Non-XLMR	X	X	O
PASD1	Non-XLMR	O	O	O
PGRMC1	Non-XLMR	X	O	O
PLS3	Non-XLMR	O	O	O
POF1B	Non-XLMR	O	O	O
RAP2C	Non-XLMR	O	X	O
RPS6KA6	Non-XLMR	O	O	O
SERPINA7	Non-XLMR	O	O	O
SLC25A43	Non-XLMR	X	O	O
SPANXB1	Non-XLMR	X	O	O
SPANXN1	Non-XLMR	O	O	O
SPANXN2	Non-XLMR	X	O	O
SPIN2A	Non-XLMR	O	O	O
SPIN3	Non-XLMR	O	O	O
SPIN4	Non-XLMR	O	O	O
STARD8	**Non-XLMR**	**X**	**X**	**X**
TAF9B	Non-XLMR	O	O	O
TBX22	Non-XLMR	X	O	O
TCEAL2	Non-XLMR	O	O	O
THOC2	Non-XLMR	X	O	O
TMEM164	Non-XLMR	O	O	O
TMEM28	Non-XLMR	X	X	O
TMLHE	Non-XLMR	O	O	O
TRO	Non-XLMR	O	O	X
UBQLN2	Non-XLMR	O	O	O
WDR44	Non-XLMR	O	O	O
ZIC3	Non-XLMR	X	O	O
ZNF275	Non-XLMR	O	O	O
ZNF711	Non-XLMR	O	O	O
ZXDA	Non-XLMR	O	O	O

“O” describes correctly classified genes. “X” described incorrectly classified genes. Only one gene was wrongly classified at all distances – shown in bold.
