# Supplementary figures and images for: A computational approach to candidate gene prioritization for X-linked mental retardation using annotation-based binary filtering and motif-based linear discriminatory analysis
Source: Biol Direct. 2011 Jun 13;6:30. doi: 10.1186/1745-6150-6-30 (PMC3142252; doi:10.1186/1745-6150-6-30)

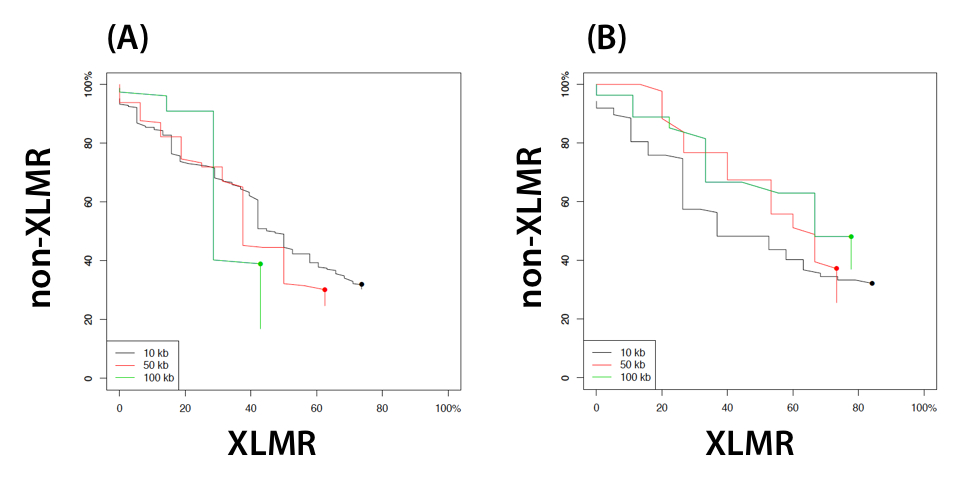

Supplement: Additional File 2 — Figure S1 - LDA classification success rates for different values of the tuning parameter τ. (A) Test set of XAR genes, with training performed on XCR genes. (B) Test set of XCR genes, with training performed on XAR genes. Leave-one-out cross-validation was utilized to calculate correct classification rates. Dots indicate optimal values of τ (see Table S6 - Additional file 1). [file 1745-6150-6-30-S2.JPEG]
